# Cognitive Decline, Neurologic Involvement, and Neonatal Crisis in *ABCC9*-Related Intellectual Disability and Myopathy Syndrome

**DOI:** 10.1212/NXG.0000000000200385

**Published:** 2026-06-10

**Authors:** Vini Nagaraj, Quentin Hugo Thomas, Paulo Ribeiro Nóbrega, Jorge Luis Rodriguez Gil, Emily Garavatti, Marcello Scala, Mariasavina Severino, Stephanie Efthymiou, Simon Amaral, Tze Chang Ng, Terrie Inder, Jian Gao, Kenneth A. Matreyek, Matheus Augusto Araújo Castro, Fernando Kok, Mauricio Takeshi Sakata, Pedro Lucas Grangeiro de Sá Barreto Lima, André Luiz Santos Pessoa, Hana Safraou, Jonathan A. Bernstein, Colin G. Nichols, Gijs van Haaften, Frederic Tran Mau-Them, Marie F. Smeland, Conor McClenaghan

**Affiliations:** 1 The Center for Advanced Biotechnology and Medicine, and the Departments of Pharmacology and Medicine, Robert Wood Johnson Medical School, Rutgers, The State University of New Jersey, Piscataway;; 2 INSERM UMR1231 Team GAD, F-21000, Université de Bourgogne Europe, Dijon, France;; 3 Department of Clinical Medicine, Universidade Federal do Ceará, Rua Alexandre Baraúna, Fortaleza - Ceará, Brazil;; 4 Department of Pediatrics, Division of Medical Genetics, Stanford University School of Medicine, CA;; 5 Department of Pediatrics, Division of Neonatal and Developmental Medicine, Stanford University, CA;; 6 Center for Neonatal Research, Children's Hospital Orange County, CA;; 7 Department of Neurosciences, Rehabilitation, Ophthalmology, Genetics, Maternal and Child Health, University of Genoa, Italy;; 8 U.O.C. Genetica Medica, IRCCS Istituto Giannina Gaslini, Genoa, Italy;; 9 Neuroradiology Unit, IRCCS Istituto Giannina Gaslini, Genova, Italy;; 10 Department of Neuromuscular Disorders, UCL Queen Square Institute of Neurology, University College London, United Kingdom;; 11 Department of Neurology, Dijon University Hospital, France;; 12 Center for the Investigation of Membrane Excitability Diseases, and Department of Cell Biology and Physiology, Washington University School of Medicine, St. Louis, MO;; 13 Department of Pathology, Case Western Reserve University School of Medicine, Cleveland, OH;; 14 Department of Neurology, University of Sao Paulo School of Medicine, Brazil;; 15 Genética Médica e Forense, Campinas, Brazil;; 16 Unité Fonctionnelle 6254 Innovation en Diagnostic Génomique des maladies rares, CHU Dijon Bourgogne, France;; 17 Department of Genetics, University Medical Center, Utrecht, the Netherlands;; 18 Department of Pediatric Rehabilitation, University Hospital of North Norway, Tromsø, Norway; and; 19 Institute of Clinical Medicine, UiT The Arctic University of Norway, Tromsø.

## Abstract

**Background and Objectives:**

The *ABCC9* gene encodes the widely expressed SUR2 subunit of ATP-sensitive potassium (K_ATP_) channels. Autosomal recessive loss-of-function variants in *ABCC9* cause *ABCC9*-related Intellectual disability and Myopathy Syndrome (AIMS). Here, we sought to compile multiple case reports from previously unidentified individuals with the primary objective of further establishing the clinical consequences of *ABCC9* variants.

**Methods:**

We combine multiple case reports with genetic diagnoses and functional tests of recombinant K_ATP_ channels.

**Results:**

We report 5 cases of AIMS, including a neonate, and a woman who presented as a sexagenarian with signs of dementia. All variants are predicted to lead to nonsense mediated decay of *ABCC9* transcripts and/or drastic truncation of SUR2. Functional tests of recombinant channels confirm that disease-associated SUR2 truncations cause a complete loss-of-function. These new cases further demonstrate the prominence of white matter abnormalities resembling periventricular leukomalacia or small vessel disease as a key hallmark of the disorder, alongside developmental delay, intellectual impairment, seizures, and fatigability. These latest findings also highlight neonatal presentation of disease, deterioration following surgical procedures, and the potential for motor and cognitive decline, which should be monitored in older individuals.

**Discussion:**

These findings provide new insights into the spectrum of pathology and natural history of AIMS. This new cohort underscores that AIMS is characterized by the combination of periventricular leukomalacia, developmental delay and intellectual disability, and muscle weakness and fatigability - and is driven by biallelic loss-of-function variants in *ABCC9*.

## Introduction

Biallelic variants in *ABCC9* have been reported in association with the emergent *ABCC9*-related Intellectual disability Myopathy Syndrome (AIMS; OMIM 619719), a disorder characterized by developmental delay, intellectual disability, white matter abnormalities, and fatigability.^1,2^ *ABCC9* encodes the SUR2 regulatory subunit of ATP-sensitive potassium (K_ATP_) channels, which are widely expressed in diverse tissues.^3,4^ K_ATP_ channels comprise octameric complexes of 4 pore-forming Kir6 (potassium conducting inwardly rectifying) subunits coassembled with 4 regulatory SUR (sulfonylurea receptor) subunits.^5-7^ Channel activity is subject to complex regulation by intracellular nucleotides, with opening being inhibited by ATP binding to Kir6 subunits and enhanced by MgADP binding to SUR subunits, thereby coupling cellular metabolism to electrical signaling.^8^

Fifteen AIMS patients have previously been reported. All harbored biallelic stop-gain or frameshift variants which were predicted to result in nonsense mediated decay of mRNA transcripts and/or major deletions or truncations in expressed SUR2 proteins. Studies of recombinant channels revealed that AIMS variants resulted in complete loss-of-function (LoF) of SUR2-dependent K_ATP_ channels.^1,2^ Here, we report 5 new biallelic patients with 5 novel variants in *ABCC9*, including a compound heterozygous individual. All variants result in premature stop codons, and functional studies demonstrate that the predicted SUR2 protein truncations result in a complete loss-of-function of recombinant K_ATP_ channels. These affected individuals present with recurrent AIMS features including developmental delay and intellectual disability of variable severity, leukoencephalopathy, and muscle weakness. Included, we report a case of progressive cognitive decline with aging in an older AIMS participant and a neonatal case demonstrating early onset seizures, cardiogenic shock precipitated by surgery, and progressive MRI changes, further expanding the phenotype of *ABCC9*-related disorders.

## Methods

### Study Participants and Genetic Analysis

Participants were identified from clinical centers in France, Brazil, and the United States. Exome (ES) or genome sequencing (GS) was performed, as described in eMethods. *ABCC9* variants are reported according to RefSeq NM_005691 (GenBank NC_000012.12), using HGVS recommendations.^9^

### Functional Characterization

For membrane potential fluorescent dye experiments, stable cell lines expressing human Kir6.2 and reference (hereafter, ref) or mutant human SUR2A were generated using the Landing Pad approach previously described.^10,11^ The human Kir6.2 coding sequence, followed by an IRES sequence, and either ref SUR2A or variant SUR2 sequences were cloned into an attB_PuroR-2A-mCherry plasmid.^10,11^ Landing pad HEK293 cells were transfected with attB_hKir6.2-IRES-hSUR2A-IRES-PuroR-2A-mCherry plasmids alongside a pCAG-NLS-Bxb1 plasmid to transiently express the Bxb1 recombinase enzyme, allowing for integration of the attB plasmid into the genomic Landing Pad site. Cells were cultured in DMEM (with 10% fetal bovine serum and 1% penicillin/streptomycin) supplemented with 2 µg/mL doxycycline and 0.5 µg/mL puromycin to drive gene cassette expression and select for integration, respectively.

After 2 weeks of culture, stable mCherry expression was observed in >90% of cells. Cells were plated into 96-well plates at 5 × 10^4^ cells/well and incubated overnight in culture media before being washed with assay buffer containing (in mM): 139 NaCl, 1 KCl, 2 CaCl_2_, 1 MgCl_2_, 10 HEPES, 10 Glucose (pH 7.4), and incubated with 80 µL assay buffer containing 1 x FLIPR Blue Membrane Potential Dye (Molecular Devices). To activate K_ATP_ channels, certain wells included 2.5 µg/mL oligomycin A, an inhibitor of ATP synthase which is expected to reduce intracellular ATP concentrations. Oligomycin A was accompanied by 10 µM glibenclamide in certain wells to inhibit K_ATP_ channels. FLIPR Blue dye was excited at 510 nm and emission recorded at 565 nm via a Biotek Neo2 plate reader. Untransfected Landing Pad cells were included as a negative control. Data were acquired from 6 independent experiments, and for each experiment, the fluorescence in the presence of oligomycin A, in the absence or presence of glibenclamide, was normalized to the fluorescence from the same cell type in assay buffer alone. Statistical significance was assessed by Mann-Whitney U tests of FLIPR dye fluorescence in oligomycin A compared with fluorescence in oligomycin A + glibenclamide for each cell type, using the Holm-Šídák method for multiple comparisons (an adjusted *p* value <0.05 was considered statistically significant).

For patch clamp analysis, Glu961LysfsTer10, Gly1090AspfsTer2, and Ser601ArgfsTer9 variants were introduced into reference human SUR2A and cloned into the pcDNA3.1(-) plasmid. HEK293 cells were transfected with 0.3 µg pcDNA3.1_mKir6.2 (GenBankTM accession no. D50581.1) and 1 µg ref or variant hSUR2A encoding sequences alongside 0.1 µg pcDNA3.1_eGFP constructs to identify transfected cells. To model heterozygous coexpression of ref and variant subunits, cells were transfected with Kir6.2 and eGFP, as above, alongside either 0.5 µg of pcDNA3.1 plasmids encoding ref SUR2A sequences with 0.5 µg of pcDNA3.1_eGFP, or 0.5 µg of ref SUR2A encoding sequences with 0.5 µg of the SUR2 variant encoding sequence cDNA.

Patch clamp electrophysiology methods were reported previously.^1^ In brief, recordings were made from HEK293 cells 36–48 hours post-transfection. Currents were recorded in response to voltage ramps from −100 to +60 mV from a holding potential of −80 mV (sampled at 10 kHz with low-pass filtering at 1 kHz). The bath solution contained (in mM): 136 NaCl, 6 KCl, 2 CaCl_2_, 1 MgCl_2_, 10 HEPES, and 10 glucose (pH 7.4 with NaOH). The pipette solution contained (in mM): 140 KCl, 10 NaCl, 1 MgCl_2_, 10 HEPES, 0.5 CaCl_2_, 4 K_2_HPO_4_, and 5 EGTA (pH 7.3 with KOH). Glass micropipettes measured resistances of 2.5–4 MΩ when filled with pipette solution. Recordings were performed at 20°C–22°C. Whole-cell currents were measured immediately after membrane rupture and for 10 minutes thereafter. Liquid junction potentials were uncorrected. Patch clamp data were analyzed with a Kruskal-Wallis omnibus test, followed by Dunn tests for pairwise comparisons.

### Standard Protocol Approvals, Registrations, and Patient Consents

Written informed consent was obtained for all living participants before inclusion in the study or approval for publication was granted from the Institutional Review Board when the family of one patient did not respond to follow-up contact.

### Data Availability

Primary data generated during this study are reported within the published article or available from the corresponding authors on reasonable request. Variants were submitted to the Leiden Open Variation Database with accession numbers, as reported in eMethods.

## Results

### Clinical Observations and Genetics

Table presents a clinical summary from this cohort and previously reported AIMS participants.

**Table:** Summary of Major Clinical Features in AIMS

Clinical features	Participant 1	Participant 2	Participant 3	Participant 4	Participant 5	This cohort (n = 5)	Total including previous cohorts^a^ (n = 20)	%
Developmental delay	Y	Y	Y	Y	N	4	19	95
Intellectual disability	Y	Y	Y	N	N	3	17	85
Fatigability	Y	Y	N	N	N	2	15	75
White matter signal alterations	Y	Y	N	N	Y	3	14	70
Lordosis/Scoliosis	N	Y	N	N	N	1	11	55
Dysmorphism	N	Y	Y	Y	N	3	13	65
Neuropsychiatric manifestations	N	Y	Y	N	N	2	11	55
Contractures	N	Y	N	N	N	1	10	50
Microcephaly	N	N	N	N	N	0	8	40
Corpus callosum hypoplasia/agenesis	N	Y	N	N	N	1	7	35
Seizures	N	Y	N	N	Y	2	7	35
Cardiac abnormalities	N	N	N	N	Y	1	4	20
Other MRI abnormalities	Y	N	N	N	N	1	3	15

a PMID: 31575858, 38217872.

*Participant 1*, a female sexagenarian, was referred to a hospital clinical genetics department regarding a progressive cognitive impairment with white matter abnormalities. She was born to a consanguineous couple (parents were first degree cousins) following uneventful pregnancy and delivery. Her medical history included global developmental delay (DD): she started walking at 3 years and started speaking even later. However, she was never referred to a pediatrician or provided with complementary tests for DD.

Learning difficulties and intellectual disability were noticed during primary school, motivating adapted schooling in a specialized institute from third grade. Despite these early difficulties, she led a quite independent life including working in an adapted environment until her forties. At this age, family members and managers at work started noticing a decline in her motor and cognitive abilities. These symptoms progressively worsened, motivating neurologic attention at the age of 60 years when she could barely walk alone, fell frequently, and could no longer hold a full conversation or answer open questions.

**Figure 1:**
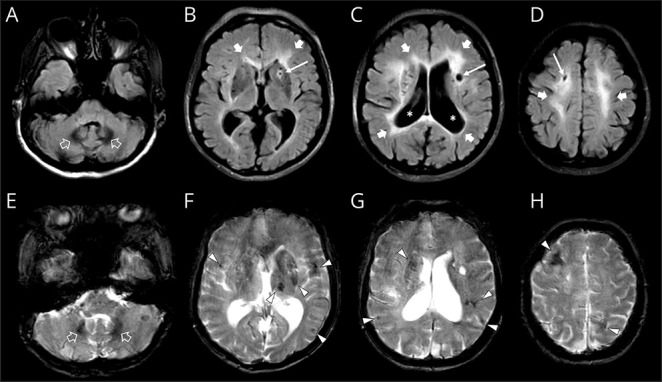
MRI Neuroimaging Findings Brain MRI from Participant 1 at 60 years, with axial Fluid-Attenuated Inversion Recovery (FLAIR) images (A–D) and gradient echo T2*-weighted images (E–F) revealing reduced periventricular white matter volume with consequent enlargement and posterior squared appearance of the lateral ventricles (asterisks). A–D Extensive signal alterations of the frontal and parietal periventricular white matter (thick arrows), also involving the left anterior temporal lobe and external capsules. Small cavitations or lacunar infarcts are noted in the cerebral white matter and basal ganglia (thin arrows). E–F Multiple areas of low T2* signal likely consistent with calcifications at the level of the subcortical cerebral and cerebellar white matter, basal ganglia, and brainstem (arrowheads). The cerebellar dentate nuclei are also likely calcified (empty arrows). Overall, these findings are consistent with a severe vascular leukoencephalopathy.

MRI of the brain (Figure 1), at age 60 years, revealed reduced periventricular white matter volume with extensive signal alterations also involving the left anterior temporal lobe and external capsules. Widened perivascular spaces were noted in the basal ganglia, associated with small cavitations/lacunar infarcts in the cerebral white matter. Finally, there were multiple areas of low T2* signal likely consistent with cerebral calcifications at the level of the subcortical cerebral and cerebellar white matter, basal ganglia, and brainstem. These findings were suggestive of a vascular leukoencephalopathy. A panel of 6 genes associated with vascular leukoencephalopathy (*APP*, *NOTCH3*, *HTRA1*, *COL4A1*, *COL4A2*, *TREX1*) failed to reveal any pathogenic variant.

Exome sequencing was performed and revealed a homozygous frameshift variant *ABCC9* (NM_020297.4; hg38) c.2881del; p.(Glu961LysfsTer10). This variant is documented in the gnomAD database (v4.1.0) at a heterozygous allele frequency of 6.2 × 10^−7^, with no homozygous occurrence noted (see eMethods). Clinical examination at the age of 61 years, using a reverse phenotyping approach, revealed global muscle atrophy, particularly marked on proximal muscles (quadriceps, deltoids), and diffuse muscle weakness, but once again predominating in girdle muscles, altogether evocative of myopathy. She also presented first-motor-neuron signs, particularly brisk deep tendon reflexes. She died in her early sixties from aspiration-related asphyxiation.

*Participant 2* is an adolescent female, born to consanguineous parents who presented with developmental regression when she was 15 months. She exhibited intellectual disability, a prominent forehead, epicanthal folds, a broad nasal bridge, a low frontal hairline with thick eyebrows, widely spaced upper central incisors, gingival hyperplasia, seizures, and behavior abnormalities. A slowly progressive gait disturbance was also apparent. Neurologic examination was marked by fatigability, scoliosis, and contractures. MRI of the brain revealed diffuse white matter signal abnormalities with corpus callosum hypoplasia. There were no obvious cardiac abnormalities, and her hearing was normal. Exome sequencing revealed a novel homozygous frameshift variant *ABCC9* (NM_020297.4) c.3269delG; p.(Gly1090AspfsTer2). This variant is documented in the gnomAD database at a heterozygous allele frequency of 5.6 × 10^−6^, with no homozygous occurrence noted (see eMethods).

*Participant 3* is a young adult female born to nonconsanguineous parents who presented with intellectual disability, strabismus, and short philtrum, high arched palate, and straight eyebrows. Neuropsychological evaluation revealed an IQ of 69. MRI of the brain was unremarkable. Baseline echocardiogram, EKG, and audiometry were normal. Creatine kinase was also normal. Participant 3 is unrelated to participant 2, but exome sequencing revealed the same homozygous frameshift variant, *ABCC9* (NM_020297.4) c.3269delG; p.(Gly1090AspfsTer2).

*Participant 4* is a female in early childhood, born to nonconsanguineous parents, who has a history of global developmental delay and impaired growth. Examination revealed small size. At age 2 years and 10 months growth parameters were: head circumference 47 cm, 17 percentile for age; weight 10.7 kg, 1 percentile for age; height: 83 cm, 1 percentile for age. Noted were ears with simplified helices, broad nasal bridge, columnar nose with full tip, arched eyebrows, and mildly downslanted palpebral fissures and sparse scalp hair. No contractures were observed. Echocardiogram at 3 weeks of age showed lateral branch artery pulmonary stenosis bilaterally, left ventricular dilation with preserved function and small patent foramen ovale (PFO). Follow-up echocardiographic imaging at 3 years old was normal with previous findings resolved. Whole exome sequencing analysis revealed a 73,292 bp homozygous deletion in *ABCC9* (GRCh37: chr12:21967577_22040869del) which encompasses exons 13 – 33, which are common to SUR2A (NM_005691.4) and SUR2B (NM_020297.4), and is predicted to result in a p.(Ser601ArgfsTer9) truncation. This variant is not documented in gnomAD.

*Participant 5* is an infant male, born at gestational age of 40 weeks to nonconsanguineous parents. At 32 hours of life, he was transferred to intensive care due to recurrent emesis and feeding difficulties and was found to have ileal atresia. On the third day of life, clinical seizure activity was confirmed on EEG to be multifocal in onset and he was given intravenous loads of phenobarbital followed by fosphenytoin for seizure control. MRI of the brain on day of life 4 revealed punctate periventricular white matter T1 hyperintensities and multifocal punctate areas of diffusion restriction in ventricular white matter, internal capsule, and thalami. He underwent surgical correction of ileal atresia on day of life 4 and 12 hours after surgery developed hypotension, bradycardia, and decreased urine output and lactic acidosis. Echocardiogram was notable for decreased biventricular cardiac function and a posterior muscular ventricular septal defect (VSD). Seizures became refractory requiring multiple loads and continued maintenance of phenobarbital and fosphenytoin. Owing to concerns over his hypotension and low cardiac output, fosphenytoin was discontinued and he was then loaded and maintained on levetiracetam. Because of ongoing seizure activity, he was given leucovorin and pyridoxine for possible metabolic epilepsy. Clinical status stabilized over 2–3 days with improvement in acidosis and cardiac function, and seizure control, and he was weaned off phenobarbital. Leucovorin and pyridoxine were discontinued after whole genome sequencing did not identify a metabolic epilepsy. Levetiracetam monotherapy was continued. Repeated MRI on day 12 of life showed progression of previous findings with worsened punctate periventricular white matter and corona radiata T1 hyperintensities with progression to involvement of putamen, thalami, and brainstem and diffusion restriction in the basal ganglia, corpus callosum, posterior limb of internal capsules, mid brain, and pons.

The participant was stable for 1 month, but because of feeding difficulties, a gastrostomy tube was surgically placed. The day following this second surgery the participant went into decompensated cardiogenic shock with lactic acidosis and required aggressive inotropic support. Echocardiogram demonstrated severely diminished systolic function in left and right ventricles. Seizures were seen about 3 days after surgery and were refractory to loads of levetiracetam and phenobarbital. Phenobarbital maintenance was restarted, and because of ongoing seizure activity, he was escalated to midazolam drip. Seizures were ultimately controlled with load of lacosamide 5 days after surgery, and he was started on lacosamide maintenance with continued phenobarbital and levetiracetam maintenance. His cardiac function slowly improved. He was weaned off midazolam drip 6 days after surgery and then phenobarbital several days later. He was continued on maintenance levetiracetam and lacosamide, but because of intermittent bradycardia, lacosamide was weaned off, with resolution of bradycardia, and he was continued on levetiracetam monotherapy. VSD was no longer visible on echocardiogram at 2 months of life and cardiac function was normal. Both surgeries involved propofol administration, and it was presumed to trigger decompensation. He was discharged at 83 days of life. The participant presented at 7 months of life with an increase in seizure activity, was found to have infantile spasms, and is currently undergoing treatment with adrenocorticotropic hormone.

Whole genome sequencing identified compound heterozygous *ABCC9* variants: a paternally inherited c.4018C>T p.(Gln1340Ter) and a maternally inherited c.1828_1829del p.(Leu610GlufsTer2) variant, consistent with a diagnosis of AIMS. The maternal variant was documented at a heterozygous allele frequency of 6.2 × 10^−6^ in gnomAD (see eMethods), and the paternal variant was not found. Incidentally, the participant has Glucose-6-phosphate dehydrogenase deficiency due to a maternally inherited pathogenic *G6PD* c.292G>A(p.Val98Met) hemizygous variant in cis with a hemizygous *G6PD* c.466A>G(p.Asn156Asp) variant of unknown significance.

### Extended Cardiovascular Characterization of an AIMS Family

**Figure 2:**
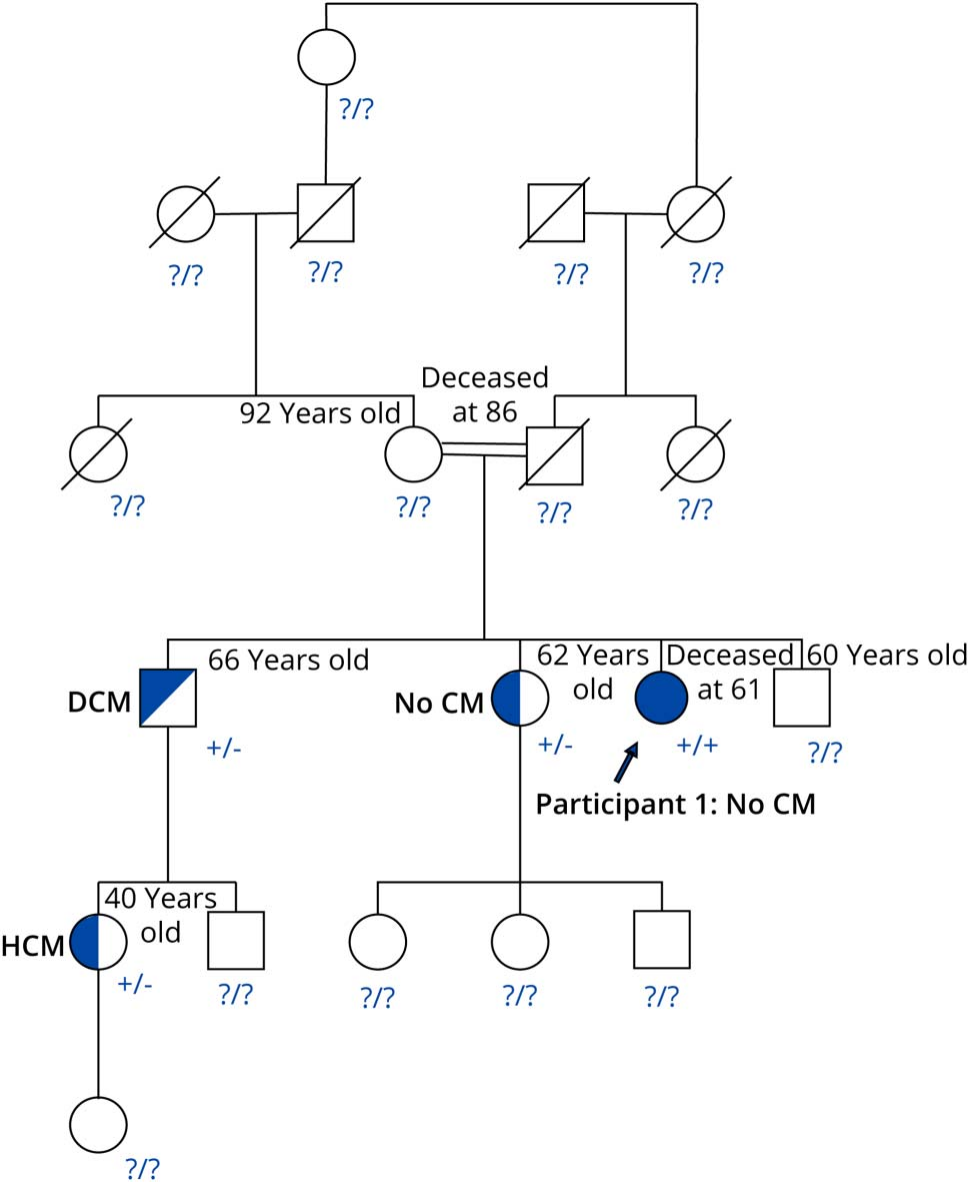
Extended Family of Participant 1 The homozygous *ABCC9* c.2881del; p.(Glu961LysfsTer10) variant was first identified in Participant 1. Familial segregation shows the variant is carried in a heterozygous sister, brother, and niece of the proband. Dilated (DCM) and hypertrophic (HCM) cardiomyopathy, respectively, are observed in the latter 2 family members.

Dilated cardiomyopathy (DCM) was previously observed in older AIMS individuals (29 and 33 years) but was not present in younger individuals.^1,2^ Subsequent follow-up revealed DCM in the carrier father of the affected older AIMS individuals.^1^ Here, we report a further familial segregation of cardiovascular phenotype in the family of participant 1 (Figure 2). No cardiac abnormalities were identified in Participant 1 before her death at age 61, but dilated cardiomyopathy was observed in a brother (66 y.o.) who was found to be a heterozygous carrier of the *ABCC9* variant. The 40 y.o. daughter of this affected brother also carries the *ABCC9* variant and presents with cardiomyopathy, although this was diagnosed as hypertrophic and not dilated cardiomyopathy. The 66-year-old sister of Participant 1 also carries the variant but displays no apparent cardiac dysfunction. Thus, while cardiomyopathy has now been observed in multiple heterozygous AIMS variant carriers it remains unclear if these variants are causal with incomplete penetrance or coincidental.

### Functional Characterization

**Figure 3:**
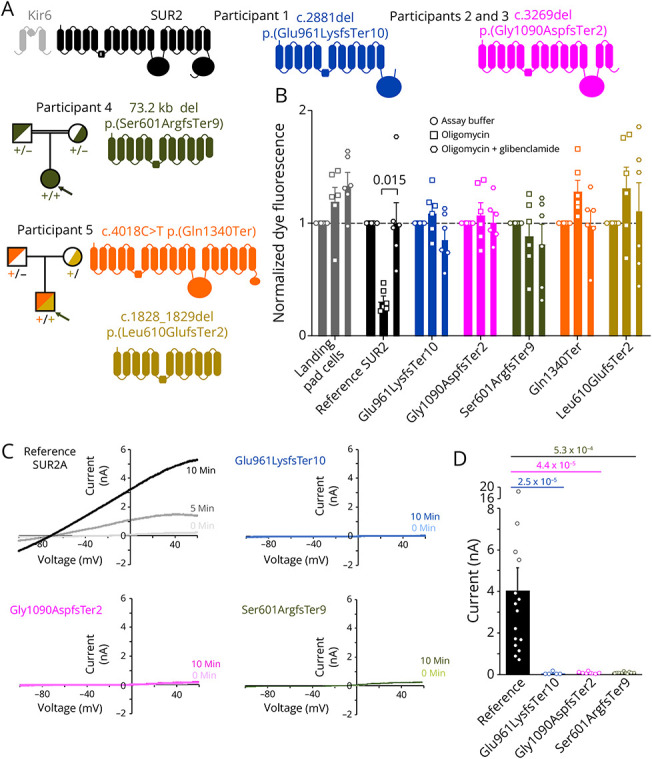
Functional Analysis of SUR2 Variants (A) Schematic diagram of Kir6 and full-length reference SUR2 (black) subunits. Predicted truncations for novel *ABCC9* variants. (B) The fluorescence from the membrane potential dependent FLIPR Blue dye was measured from Landing Pad HEK293 cells stably expressing reference or AIMS variant SUR2A, alongside Kir6.2. Dye fluorescence in the presence of oligomycin (squares) or oligomycin and glibenclamide (hexagons) was normalized to the fluorescence observed in their absence, in assay buffer alone. Data shown as mean ± SEM from 6 independent experiments. *p* value from Mann-Whitney *U* test comparing normalized fluorescence in oligomycin in the absence and presence of glibenclamide, adjusted by the Holm-Šídák method for multiple comparisons, is displayed for significant differences. (C) Example whole cell current traces from HEK293 cells transiently transfected with Kir6.2 and reference (ref) SUR2A (black, top left) or SUR2A[Glu961LysfsTer10] (blue, top right) or SUR2A[Gly1090AspfsTer2] (magenta, bottom left) and SUR2A[Ser601ArgfsTer9] (green, bottom right). (D) Summary of whole cell currents recorded at 0 mV 10 minutes after break-in to the whole cell configuration. *p* values from pairwise Dunn's Test following Kruskal-Wallis tests are displayed.

To assess functional effects of all variants, Landing Pad HEK293 cell lines stably expressing Kir6.2 with reference (ref SUR2A) or variant SUR2 were generated. Figure 3A shows the expected truncations resulting from AIMS associated variants. The membrane potential-dependent FLIPR Blue fluorescent dye was used to assess membrane hyperpolarization, an expected consequence of K_ATP_ activation. Ref and variant expressing cells were incubated with the ATP synthase inhibitor oligomycin A to reduce intracellular ATP levels and relieve channels from inhibition. This led to the expected decrease in dye fluorescence for ref expressing cells, which was reversed by the K_ATP_ inhibitor glibenclamide (Figure 3B). By contrast, cells expressing AIMS associated variants did not exhibit hyperpolarization on oligomycin A incubation, indicating a loss of K_ATP_ channel function.

**Figure 4:**
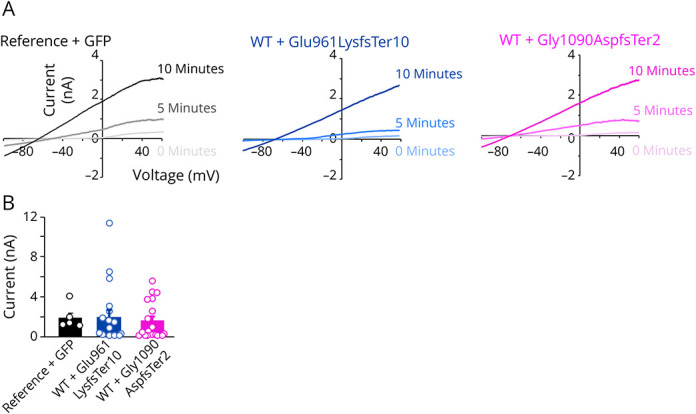
Functional Analysis of Mixed Reference and Variant SUR2 (A) Example traces of experiments to mimic heterozygous co-expression of ref and variant subunits. *Left*—50% of ref SUR2A supplemented with cDNA for GFP. *Middle*—50% ref and 50% SUR2A[Glu961LysfsTer10] cDNA. *Right*—50% ref and 50% SUR2A[Gly1090AspfsTer2] cDNA. (B) Summary of whole cell currents recorded at 0 mV 10 minutes after break-in to the whole cell configuration. Bars show mean ± SEM.

To confirm, we tested functional expression of select variants using patch clamp electrophysiology. Whole-cell recordings revealed the development of large potassium conductances in cells transfected with Kir6.2 plus reference SUR2A in a time dependent manner as inhibitory intracellular ATP is diluted by the ATP-free solution in the patch pipette. In stark contrast, only small endogenous potassium currents typically observed in HEK293 cells were seen in cells transfected with Kir6.2 plus Glu961LysfsTer10, Gly1090AspfsTer2, or Ser601ArgfsTer9 SUR2 variants (Figure 3, C and D). To determine whether variant subunits have a dominant-negative impact on K_ATP_ function, we cotransfected cells with reference and variant cDNA, each at 50% of the amount used for single transfections. As shown in Figure 4, A and B, coexpression of variant subunits with reference SUR2A did not significantly reduce K_ATP_ currents beyond the trend toward lower currents attributed to the reduced amount of ref SUR2A alone. This suggests that these variant subunits do not exert major dominant-negative effects in this recombinant expression system.

## Discussion

We report 5 individuals with biallelic variants in *ABCC9* that are predicted to cause frameshifts and nonsense-mediated decay of mRNA or major truncations in any translated SUR2 protein. These variants are either not documented or found at low allele frequencies (ranging from 6.2 × 10^−7^ to 6.2 × 10^−6^ of heterozygous individuals) in the gnomAD database, with no previous identification in homozygous individuals (see eMethods). Studies of recombinant channels reveal that these truncations abolish K_ATP_ channel activity. Affected individuals display key clinical features previously observed in AIMS patients, including developmental delay, intellectual disability, and leukoencephalopathy. These features are emerging as hallmark characteristics of AIMS and are observed alongside incidences of myopathy, strabismus, dysmorphic features, and epilepsy, in this and previous cohorts.^1,2^ Although hundreds of variants in *ABCC9* of unknown clinical consequences have been identified in heterozygous individuals, very few homozygous cases are documented, and we document the first known compound heterozygous case. Our findings confirm previous reports of biallelic LoF variants causing AIMS.

Owing to the small patient numbers identified to date, the natural history of AIMS remains poorly understood. We report on a 60-year-old woman (participant 1) who presented with cognitive and motor decline beginning when a quadragenarian. Neuroimaging findings from participant 1 reveal an advanced-stage vascular leukoencephalopathy involving the external capsule and anterior temporal lobe. Multiple dilated perivascular spaces and cavitations resembling lacunar infarcts were found in the basal ganglia and cerebral white matter. In addition, several areas of possible mineral deposition were noted in the subcortical white matter, basal ganglia, brainstem, and cerebellum. Of note, white matter changes involving anterior temporal lobes and/or external capsules and brain calcification were previously reported in other patients with *ABCC9* LoF variants.^1^ By comparison with the white matter changes in these younger patients, the findings in Participant 1 might signal a changing neuroimaging pattern along the course of disease from a leukodystrophy resembling periventricular leukomalacia with few scattered calcifications to a more severe vascular leukoencephalopathy with anterior temporal and external capsule involvement and calcifications.

Of interest, similar white matter abnormalities have been previously described in genetic vascular leukodystrophy disorders, including CADASIL and COL4A1/A2-related disorders.^12-15^ K_ATP_ channels are expressed throughout the vasculature, as well as in certain neuronal populations and astrocytes, and these clinical findings are consistent with mounting evidence that SUR2-dependent K_ATP_ channels are critical for cerebral vasculature function.^16-19^ Loss-of-function of SUR2 likely impairs cerebral perfusion which could cause ischemic events that drive white matter abnormalities.

This case expands the phenotypic spectrum of *ABCC9*-related disorders and suggests that age-related cognitive decline may be a significant component in the disease course. Further follow-up of AIMS patients will reveal whether this is a conserved feature of the disorder. Of note, expression quantitative trait locus (eQTL) variants in *ABCC9* have previously been associated with hippocampal sclerosis of aging,^20-22^ suggesting that *ABCC9*/SUR2 might play a key role in protection against age-related neurologic decline. More generally, the progression from early developmental delay to progressive cognitive decline observed in Participant 1 might reflect a broad emerging phenomenon in which early life DD can progress to cognitive decline in aging revealed by individuals born decades ago now (re)entering the health care system with dementia.

We also report an early-life AIMS case (participant 5), with neonatal progression of MRI findings across 8 days of early life. This participant experienced neonatal onset of seizures alongside the common finding of periventricular hyperintensities, but additionally, displayed diffusion restriction involving both white matter and deep grey structures. He experienced cardiogenic shock after surgery and, after recovery, exhibited progression of neuroimaging abnormalities on repeat MRI. Diffusion restriction can be seen in the setting of hypoxic injury, but importantly, his findings were not a typical pattern of hypoxic injury such as watershed injury due to hypoperfusion which can be seen in the setting of cardiogenic shock. It is unclear if the cardiogenic shock precipitated this worsening of MRI findings or if cardiogenic shock and MRI findings represent an exacerbation of underlying pathology attributed to *ABCC9*-related disorders.

A second episode of cardiogenic shock also occurred after surgical procedure with anesthesia using propofol. Both episodes were associated with lactic acidosis and worsening of seizures. It is not known whether propofol induced the cardiogenic shock in this case, but as (1) propofol has been associated with increased risk of bradycardia and has been shown to inhibit cardiac L-type calcium channels,^23-25^ and (2) another calcium channel blocker, verapamil, which can also provoke bradycardia causes sudden death in mouse models of AIMS,^26^ careful consideration should be taken before the administration of drugs with calcium channel blocking activity to AIMS patients.

Notably, 4 of the 6 patients described in our original identification of AIMS^2^ had undergone general anesthesia with propofol at ages ranging from 5 years to adulthood without noted adverse effects. These individuals all presented with the same homozygous splice-site variant and so it is possible that the degree of molecular dysfunction could be different to Participant 5, who has compound heterozygous variants, or that the age past infancy at the time of exposure could affect responses. Of interest, one patient from this earlier report (patient 1-1) received fosphenytoin infusion for a series of epileptic seizures at age 17 years, resulting in severe bradycardia and hypotension and a short loss of consciousness. Phenytoin, the active metabolite of fosphenytoin is thought to primarily mediate anticonvulsant effects via sodium channel blockade but has also been shown to block cardiac calcium channels.^27^ Fosphenytoin was administered to participant 5 due to worsening of seizures in setting of cardiogenic shock, and it was unclear if the medication negatively impacted clinical status. Lacosamide resolved seizures in participant 5 but was discontinued as maintenance therapy due to the possibility of lacosamide causing bradycardia. Lacosamide has a similar mechanism of action to fosphenytoin which includes sodium channel blocking effects.^28^ Levetiracetam was chosen for maintenance therapy in participant 5 due to its alternative mechanism of action, impacting synaptic release vesicle rather than direct modulation of channels.^28^ Further study is required to determine the potential risks of neuromodulating medications such as propofol and antiseizure medications in the context of SUR2 loss-of-function. This should include the screening of clinically used antiseizure drugs for adverse cardiovascular effects in SUR2-deficient animal models. It is important that SUR2 is a critical component of cardiac K_ATP_ channels, which activate in conditions of elevated workload or metabolic compromise to protect the myocardium. Thus, loss of SUR2-dependent K_ATP_ channels is expected to alter cardiac resilience in responses to stressors and could have complex, unpredictable influence on cardiac-acting drug effects.

Although seizures have been seen in 7 AIMS patients (Table), the development of seizures may be multifactorial. Participant 5 had seizures in the setting of MRI abnormalities with diffusion restriction which suggests acute injury. Seizures in others with AIMS does not seem to correlate with acute neurologic injury. Seizures have previously been documented at 6 weeks old without recurrence, in the setting of neurologic coma events at 1 and 2 years old, and in 15-month-old with polymicrogyria, a developmental brain malformation commonly associated with seizures.^1^ Seizures have been seen in teenagers both with and without EEG findings, suggestive of underlying risk for epilepsy. Participant 5 went on to develop infantile spasms, a type of epileptic encephalopathy that can be seen in association with genetic conditions, structural abnormalities or prior brain injury.

Participant 5 also exhibited ileal atresia, which does not appear to have been reported previously in individuals with AIMS. Unlike duodenal atresia, which can be associated with chromosomal abnormalities, there are no known genetic associations for ileal atresia, which are most commonly the result of vascular insult *in utero*.^29^ K_ATP_ channels are expressed in vascular mural cells throughout the body and thus is it possible that loss of K_ATP_ function in the mesenteric circulation might cause vascular insult triggering intestinal developmental abnormalities.^30^ If so, this would implicate prenatal K_ATP_ channel dysfunction in AIMS pathology. K_ATP_ channels also regulate intestinal smooth muscle, and overactivity reduces gut motility in the SUR2 gain-of-function syndrome, Cantú Syndrome.^31^ Thus, it is possible that K_ATP_ loss in GI smooth muscle could cause pathology in AIMS, which should be monitored for. Pulmonary artery stenosis was observed in participant 4, which could also arise from abnormal vascular development or excess tone due to of K_ATP_ channel loss in smooth muscle or the endothelium.

Of interest, sparse hair is observed in participant 4 here and has previously been observed in other AIMS patients. Again, this contrasts with Cantú Syndrome, which is prominently associated with excessive hair growth.^32^ K_ATP_ channel loss-of-function in AIMS is expected to increase cellular excitability and thereby increase activation of voltage-gated calcium (Ca_V_) channels in cells in which the channels are coexpressed, including hair follicle cells. Notably, baldness at birth and slow hair growth is also observed in the Ca_V_1.2 gain-of-function channelopathy, Timothy Syndrome.^33,34^

Finally, as previously noted, heterozygous *ABCC9* variant carriers do not display obvious neurologic or myopathic impairments. Of interest, however, cardiomyopathy was noted among 2 carriers in the extended family of Participant 1, although this was not completely penetrant as a female carrier showed normal cardiac function. This adds to a previous report of DCM in the carrier father of 2 AIMS patients who also displayed cardiac systolic dysfunction.^1,2^ Partial loss-of-function variants in *ABCC9* have previously been reported in isolated case reports of DCM patients, and thus, it is possible that incomplete SUR2 loss-of-function can lead to cardiac restricted pathology with incomplete penetrance.^35,36^

Taken together, this study strengthens and expands the association of biallelic loss-of-function *ABCC9* variants with AIMS, revealing neonatal complications and progressive cognitive impairment with aging. Serial imaging should be considered to evaluate disease progression. Future work should include longitudinal tracking of AIMS patients to map the natural history of the disorder, and the possible adverse effects of drugs that block calcium channel activity should be further explored.

Given this growing AIMS cohort, we recommend that the constellation of periventricular leukomalacia, developmental delay and intellectual disability, and muscle weakness and fatigability in patients should prompt investigation for an *ABCC9*-related disorder.

## Glossary

AIMS: ABCC9-related Intellectual disability Myopathy Syndrome
DCM: Dilated cardiomyopathy
DD: developmental delay
K_ATP_: ATP-sensitive potassium
LoF: loss-of-function
VSD: ventricular septal defect
